# Neurological and Vascular Behçet’s Disease in a Young Male without Classic Triad of Behçet’s Disease: A Case Report and Literature Review

**DOI:** 10.31138/mjr.240723.nav

**Published:** 2024-01-29

**Authors:** Gaurang Deshpande, Ritasman Baisya, Phani K Devarasetti, Liza Rajasekhar

**Affiliations:** Department of Clinical Immunology and Rheumatology, Nizam’s Institute of Medical Sciences, Hyderabad, India

**Keywords:** NBD, cascade sign, vascular BD, case report

## Abstract

**Introduction:**

Neuro-Behçet’s disease (NBD) is an uncommon presentation in Behçet’s disease (BD) with severe course and worse prognosis. Both vascular and NBD presentation without the classical triad of BD in a single patient is rarely reported.

**Case Presentation:**

Here a 48-year-old male had an extensive aortic aneurysm eroding vertebra for which he was diagnosed as vascular BD. Two years later, he was presented with a severe headache and cerebrovascular accident, his brain imaging showed hyperintensity in the right thalamus, basal ganglia, temporal lobe, and internal capsule, suggesting the ‘cascade sign’ of NBD. Surprisingly, he never had oral or genital ulcers or skin and eye involvement. He had a good response to infliximab.

**Conclusion:**

Clustering of BD phenotype is an emerging area of interest. It is hypothesised that severe phenotype of vascular and parenchymal NBD can happen in the same patient owing to similar underlying pathology. This case is unique due to its severe phenotype with no features of the typical triad of BD.

## INTRODUCTION

Behçet’s disease (BD), a multisystemic variable vessel vasculitis, is characterised by a triad of recurrent oral and genital ulcers and uveitis.^1,2^ In addition it commonly presents with other manifestations such as skin lesions, arthritis, thrombophlebitis, arterial aneurysms, and deep vein thrombosis (DVT). Vascular involvement known as vascular BD has also been reported in the literature even in the absence of the classic triad.^3^ Neurological involvement^4^ referred to as neuro-Behçet’s disease (NBD) is a rare occurrence with variable frequency (5–14%). It is predominantly observed in Mediterranean countries.^5^

Data on NBD in the Indian population is limited. The occurrence of NBD and vascular BD in a single patient without any components of the classic triad of BD is an extremely rare presentation. In this case we present a patient with vascular BD who experienced a major cerebrovascular accident (CVA) as the primary manifestation of NBD. The patient showed significant improvement upon receiving biological therapy.

## CASE PRESENTATION

**Figure 1.**
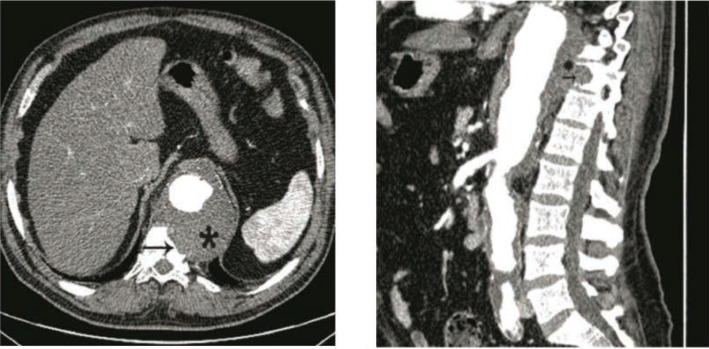
Magnetic resonance (MR) aortogram showed gross increase in the size of descending aorta aneurysm [7.4 X 6.5 X 6 cm] which was eroding the D8 vertebral body (asterisks and arrow).

A 48-year-old male was admitted with complaints of sudden onset weakness in his left upper and lower limbs for one week. He also experienced slurring of speech and deviation of the angle of his mouth to the right side. Additionally, he reported having a throbbing holocranial headache for three days along with blurred vision in both eyes, more pronounced on the right side. It is important to note that two years before this current presentation the patient experienced left supra-clavicular swelling that lasted for four months. During that time, he also had central chest pain, shortness of breath, and significant weight loss. The patient sought treatment for tuberculosis elsewhere, but unfortunately there was no response to the empirical treatment he received. Ultrasound of swelling showed that it was probably from vascular origin followed by contrast enhanced computed tomography of chest, which was suggestive of saccular aneurysm of left subclavian artery with thrombosis, aneurysm of descending thoracic aorta (6.5*5.5*5.3 cm) at dorsal vertebra 6 & 7 (D6, D7) level, and coeliac artery aneurysm. He also had a history of superficial thrombophlebitis three years back, predominantly involving lower limb. There was no history of recurrent oral and genital aphthous ulcers. Investigations showed high erythrocyte sedimentation rate (ESR) (70mm/hr) and the Pathergy test was positive. He was diagnosed as vascular BD and initiated on 1 mg/kg equivalent oral prednisolone and monthly intravenous cyclophosphamide pulses as per 2018 update of the EULAR recommendations for the management of BD. Six months after initiation of therapy, the patient developed new onset chest pain associated with shortness of breath and increasing weight loss and diminished pulse volume in left upper limb. With clinical suspicion of aortic dissection, magnetic resonance (MR) aortogram was done which gross increase in the size of descending aorta aneurysm [7.4*6.5*6 cm] which was eroding the D8 vertebral body (**Figure 1**). Patient underwent emergency thoracic endovascular aortic repair (TEVAR) and was advised the need of hiking up the immunosuppression to infliximab but could not be started in view of financial constraints. Patient was continued on monthly cyclophosphamide, a total of 9 doses (cumulative dose 6.5 grams) and then lost follow up. One year later, he presented with the above neurological symptoms.

Upon examination, he had a slender physique. All pulses were palpable without any palpable thrill or audible bruit. There were no discrepancies in blood pressure between his limbs. He was conscious, oriented. Cranial nerve examination showed motor aphasia, reduced visual acuity in both eyes (right>left) with left side upper motor neuron facial nerve palsy. Motor examination revealed left sided spastic hemiparesis and extensor plantar reflex. The remaining findings from the systemic examination were deemed insignificant.

**Figure 2.**
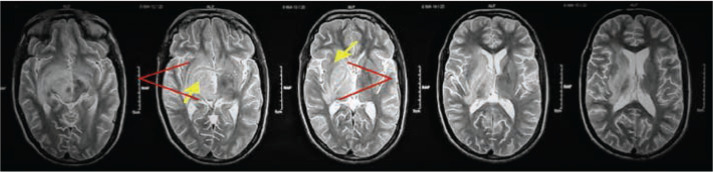
Axial fluid-attenuated inversion recovery MRI showing extensive hyperintensity with mild mass effect involving the right temporal lobe, basal ganglia, internal capsule, and thalamus suggesting the classic/cascade sign of NBD (red bracket showed extension while yellow arrow indicates the hyperintense lesion.

**Figure 3.**
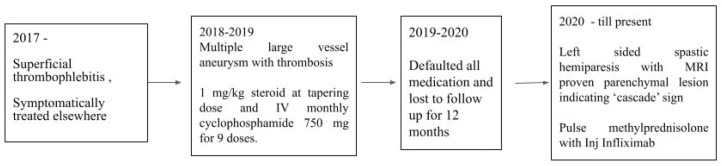
Flow diagram of timeline of different major events in the case.

Considering the high possibility of NBD, magnetic resonance imaging of brain with contrast was done which showed T2 and fluid attenuated inversion recovery (FLAIR) hyperintensities in right side of corona radiata, basal ganglia, midbrain, optic radiation, and inferior temporal cortex suggestive of ‘cascade sign’ (**Figure 2**). Cerebrospinal fluid analysis was normal. Patient was treated with intravenous methylprednisolone pulse (750mg per day) for 7 days and later started on 1 mg/kg oral steroid at tapering dose along with intravenous infliximab (5 mg/kg/dose) on 0, 2, 6 week schedule followed by every 8 weeks and azathioprine 100 mg / day. In follow-up the patient had significant improvement in motor and visual symptoms. Informed consent was taken from the patient for publication of this unique case. The flow diagram (**Figure 3**) depicts important times regarding new manifestations and therapeutic options in this case. **Table 1** represents the dose and duration of all prescribed medicines during the treatment course.

**Table 1.** Doses and duration of prescribed drugs in this case.

**Year**	**Main symptoms**	**Medicine prescribed with dose**	**Duration**
2017	Superficial thrombophlebitis	Symptomatic treatment elsewhere	
2018	Arterial aneurysm and thrombosis	1. 1 mg/kg equivalent oral Prednisolone with tapering dose 2. Inj. Cyclophosphamide 750 mg monthly once	9 months (Cumulative dose 6.5 grams )
2020	Parenchymal Neuro-Behçet’s hemiparesis	1. IV Methylprednisolone 750 mg 5 doses 2. 1 mg/kg steroid at tapering dose 3. Infliximab 5mg/kg at 0,2,6 weeks followed by every 8 weeks; escalated to 8 mg/kg after 12 weeks 4. Azathioprine 100 mg/day	Medicines 2,3,4 are continued till date

## DISCUSSION

Here we present a follow-up case of BD with extensive vascular involvement. Due to non-compliance with treatment, the patient subsequently presented with a cerebrovascular accident which is a rare manifestation of parenchymal NBD. This case presented a unique scenario as it involved the association of vascular BD and parenchymal NBD without the presence of typical BD features. However, it is important to note that the occurrence of such an association in a single case report does not definitively prove a causal relationship as it could potentially be due to chance.

BD is a complex and heterogeneous disease with a wide spectrum of manifestations ranging from subtle muco-cutaneous involvement to life-threatening vascular or neurological complications. While oral ulcer, genital ulcer and uveitis are commonly recognized as the classic triad of BD many patients present with additional manifestations that can lead to a severe disease course. The phenotypic classification of such a heterogeneous disease is of great interest to scientists. Seyahi^6^ proposed six phenotypes of BD which include skin-mucosa involvement, joint involvement, vascular involvement, eye involvement, parenchymal neurological involvement, and gastrointestinal involvement. These different phenotypes help in better understanding and categorising the diverse clinical presentations of BD. By classifying patients into these specific phenotypes researchers and clinicians can gain insights into the pathogenesis prognosis and treatment strategies for each phenotype.

NBD was initially documented in 1941 by Knapp^7^ and the term “neuro-Behçet’s syndrome” was introduced by Cavara and D’Ermo in 1954.^8^ The reported occurrence of neurologic involvement among patients with Behçet’s disease varies from 4% to 49% typically occurring more than 10 years after the onset of Behçet’s disease.^9,10^ In 2014, Kalra et al. proposed diagnostic criteria for NBD^11^ which were later adapted and modified by the International Neuro Behçet’s Advisory Group. These criteria are recommended for the diagnosis of NBD. According to this consensus the diagnosis of NBD is categorised as ‘probable’ or ‘definite’ based on the probability of BD according to the International Study Group (ISG) criteria, the presence of characteristic neurological signs and symptoms, supportive evidence from neuroimaging and cerebrospinal fluid (CSF) analysis. However, neither of these criteria have been validated. Interestingly, our patient did not qualify as per the International Consensus Recommendation (ICR) criteria (2014) for diagnosis of BD even after the classical vascular and neurological involvement, in view of lacking classical muco-cutaneous and ocular features.

### Search Strategy

After encountering the case of parenchymal NBD we started exploring its presentation and treatment approaches as described in the literature. Additionally, we aimed to investigate the potential association between vascular involvement and NBD. To achieve this, we conducted a comprehensive updated search of various databases including ‘PubMed’, ‘PMC’ ‘Embase’, ‘Web of Science’, ‘IndMed’, ‘MEDLINE’ and ‘Neurol India’ until August 2023. Using key MeSH terms such as ‘NBD’, ‘vascular Behcet’, ‘Behcet’s Disease’, and ‘imaging in NBD’, we focused on retrieving peer-reviewed original articles, review articles and case series that could provide valuable insights into the topic.

### Studies on NBD in the Literature

Many retrospective studies conducted in Mediterranean and Far East countries have focused on analysing the clinical presentation of parenchymal NBD. These studies have consistently reported that the most common initial manifestations of NBD include headache and pyramidal signs. However, it is worth noting that other common presentations of parenchymal NBD have also been observed. Optic nerve involvement is another frequently observed clinical presentation of parenchymal NBD. Additionally other cranial neuropathies along with cerebellar signs, cognitive dysfunction, psychosis, seizure, dysarthria, and movement disorders have been identified as common manifestations of the disease.^10,12^

A South Korean database study^12^ revealed that pyramidal signs were the most prevalent manifestation of parenchymal NBD accounting for 52% of patients. Additionally, headache was observed in 45 patients (45.9%) while 31 patients (31.6%) exhibited fever. The study also found that symptoms or signs associated with brainstem involvement were commonly observed. Specifically, dysarthria was reported in 42 patients (42.9%), diplopia in 25 patients (25.5%), and ataxia in 22 patients (22.4%). Nilufer Kale et al. described 17 cases of BD, the neurologic presentations included - parenchymal involvement in 12 (70%), extraparenchymal involvement/venous thrombosis in 5 (30%), seizure disorder in 2 (12%), and psychiatric problems/depression or anxiety in 5 (30%) patients. They characterised ‘Behçet headache’ as subacute, moderate to severe with unilateral localisation and throbbing quality accompanied by nausea, vomiting, and aggravation upon awakening.^13^ Our case is also presented with parenchymal involvement with pyramidal symptoms, headache, and visual blurring.

Brain stem involvement can occur as an isolated lesion or in combination with cerebellar, basal ganglia, cerebral cortex, and spinal involvement called ‘brainstem plus’. Isolated spinal cord involvement is extremely rare.^9^ Western case series found that presentation with neurological features was commoner in their patients (23%) than Middle Eastern series (3–10%). Seizures (27%) were likewise commoner (0–5%), as was optic neuritis (9% compared with 1–2%).^14^ Two patients developed movement disorders (chorea and parkinsonism), which have only been rarely reported. A monocentric study from Tunisia included 121 NBD patients.^15^ Parenchymal involvement occurred in 74 patients (61%). Among them 26 (21.4%) presented with brainstem involvement, 24 (19.8%) with hemispheric involvement, and 2 (1.6%) with spinal cord involvement.

Ertugrul Cagri Bolek et al.^16^ reported about 77 NBD patients (Definite NBD = 61, possible NBD = 16). Brainstem (72.9%) was the most frequently affected parenchymal area, followed by cerebellum (43.8%) and diencephalon (37.5%). Neurological involvement is seen about 5 years after the diagnosis of BD and More than half of the patients with acute onset parenchymal NBD had only one attack. Biologic agents (Interferon-alpha and anti-TNF agents) were used in most patients. Our patient had a median duration of the disease for two years with acute onset parenchymal NBD with good response to TNF inhibitors.

### Imaging in parenchymal NBD

Afshin Borhani-Haghighi et al.^17^ observed that the dominant acute NBD lesions seen in brain magnetic resonance imaging (MRI) are mesodiencephalic lesions. These lesions often exhibit a distinct pattern of extension from the thalamus to the midbrain giving rise to what researchers refer to as the ‘cascade sign’. In our particular case, our patient also exhibited a similar ‘cascade sign’ on their MRI scan.

Siamak Farahangiz^18^ conducted a study on patients with parenchymal NBD and reported that the most commonly affected areas were the periventricular and superficial cerebral white matter followed by the midbrain and pons. Among the patients with parenchymal involvement 24% exhibited lesion extension while 14% showed contrast enhancement. Park KS^19^ also conducted a similar study and observed that most parenchymal lesions appeared as high signal intensity on T2-weighted images. These lesions were predominantly found in the midbrain, pons, basal ganglia, and white matter. In agreement with their findings our patient also exhibited extensive involvement in the mesodiencephalic region displaying high signal intensity on T2-weighted images as well as contrast enhancement.

Ahmed Serkan Emekli^20^ included 66 non-standardised acute relapse MRIs of 55 patients with parenchymal NBD. The most frequently affected parenchymal structures were the rostral pons, mesencephalon, and diencephalic region. Patients with positive pathergy tests were more likely to present right hemisphere involvement. Our patient having Pathergy test positivity presented with a right sided lesion.

### NBD in Indian Studies

Sharma et al^21^ presented a report of three clinically distinct cases of NBD disease from North-west India. The first case was a 35-year-old male with a history of recurrent oral and genital ulcer, acute anterior uveitis, positive pathergy test and superior sagittal sinus thrombosis. In the second case, a 39-year-old male with history of oral ulcer presented with headache, left 9^th^, and 10^th^ cranial nerve palsy with left cerebellar ataxia. MR imaging (MRI) brain T2 and fluid attenuated inversion recovery revealed hyperintensities in the right pons, left midbrain, and middle cerebellar peduncle without contrast enhancement. The third case was a 29-year-old female with recurrent orogenital ulcer and anterior uveitis presented with headache and episodes of generalised tonic–clonic seizure. MRI brain revealed multiple T2 hyperintensity in the subcortical and pericallosal region with pachymeningitis. These cases suggest diversity of the neurological presentation in NBD with sub-acute to chronic presentation. In another report by Raghavendra et al^22^ a 29-year-old man, presented with headache, lateral rectus palsy, right frontotemporal subdural effusion, and a right ponto-medullary lesion, hyperintense on T2 weighted images. Patient was treated with 1 mg/kg steroid resulting in partial to full recovery in all the disease manifestations. Recently Magale et al. published 12 cases of NBD^23^ from India with pyramidal symptoms being most common, thalamus involvement was seen in all cases. Systemic involvement was in six patients, none had peripheral arterial aneurysm or DVT. In our case there was an acute presentation with extensive parenchymal involvement including brainstem, basal ganglia, optic radions with past history of vascular aneurysm and thrombosis.

### Association of NBD and vascular BD

In a study by Youness Habtany et al.^24^ it was reported that NBD could be presented as a mixed vascular and parenchymal pattern, in that study those patients had cerebral venous sinus involvement along with parenchymal NBD. Intracranial arterial aneurysm was also rarely reported in NBD. A recent cluster analysis study from China among 860 BD patients, they identified five distinct clusters - a skin and mucosa subtype, an articular subtype, a gastrointestinal subtype, a uveitis subtype, and a cardiovascular subtype with CNS involvement.^25^ They proposed that cardiovascular and parenchymal NBD might share common underlying pathogenesis with severe presentation. In this case report, the patient had both vascular and parenchymal NBD which again proves their observation. Houman et al. study of 121 NBD patients showed that association of arterial aneurysm and deep vein thrombosis were commoner in NBD compared to other types of BD.^15^

Our patient showed partial response to treatment with pulse steroid therapy and greater response was seen with infliximab therapy on follow up. In case of refractory or severe presentation TNF inhibitors have shown good results, infliximab being the most studied molecule.^26^ Our patient received azathioprine^27^ along with a high dose steroid for parenchymal NBD. However, considering the disease progression in the past despite the treatment with cyclophosphamide, extensive vascular disease and new onset CNS involvement infliximab as add-on therapy was considered.

**Table 2** shows the area of CNS involvement in two case series of parenchymal-NBD.^28,29^ **Table 3** depicts comparison of different case series and original articles on NBD across the World.^30–34^

**Table 2.** Localisation of CNS involvement in two case series of parenchymal-NBD.

**Turkey (Koçer N et al.)** ^**28**^	**Frequency**	**China (Dong Yan et al.)** ^**29**^	**Frequency**
**N:65**		**N:42**	

Mesodiencephalic junction	30 (46%)	Brainstem involvement	23 (54.8%)
Pontobulbar region	26 (40%)	Isolated brainstem	10 (23.8%)
Hypothalamic-thalamic region	15 (23%)	Brainstem plus	13 (31.0%)
Basal ganglia	12	Hemi-Cerebral involvement	22 (52.4%)
Telencephalon	5	Spinal cord involvement	5 (11.9%)
Cerebellum	3	Cervical cord	4 (9.5%)
Cervical cord	3	Thoracic cord	1 (2.4%)
		Cerebellum	4 (9.5%)
		Localisation not possible	3 (7.1%)
		More than two sites	13 (31.0%)

**Table 3.** A detailed review of different NBD studies.

**Name of the studies**	Shahien et al.^10^	Seung Woo Kim et al.^12^	Nilufer Kale et al^13^	Houman et al.^15^	A Riera-Mestre et al.^30^	Ideguchi H et al.^31^	Talarico et al.^32^	Sbai et al.^33^	C Sharma et al.^21^	Noel N et al.^34^	Present study
**Population**	16	110 - 98 parenchymal NBD	17	121	20	54	44	161	3	100	1
**Ethnicity**	Middle Eastern or North African origin	South Korean	Turkey	Tunisia	Spain	Japan	Italy	France	North India	Europe, North Africa, others	India
**Mean age of presentation**	31.6	37.6±10.6	41+ 11	29.7	36.3	36.9+.11.9	25	32	34.33	33	48
**Clinical Features**	Cognitive impairment (19%), severe headache (12.5%), convulsive disorders (12.5%), dural sinus thrombosis (6%), optic atrophy (19%) and hemispheric syndrome (19%), severe brainstem lesions (12.5%)	Parenchymal NBD (98) pyramidal symptoms headache, dysarthria, paraesthesia	Parenchymal 12, seizure 2, anxiety 5	Parenchymal (74) - brain stem, hemisphere, spinal cord	Parenchymal NBD - 16, brainstem 9	Acute parenchymal - 38, chronic progressive parenchymal - 15	Parenchymal NBD -35, brain stem - 16	Parenchymal NBD - 109	Sinus thrombosis, ataxia, headache, seizure	78 - acute NBD, 37 progressive NBD Headache - most common involvement	Parenchymal NBD as stroke and aphasia
**Imaging**	Parenchymal lesion in one patients CT scan	MRI in 87 patients brain stem, white matter, basal ganglia, thalamus	All patients - periventricular, subcortical, brainstem lesions or dural sinus thrombosis	Abnormal cranial MRI 74/92	Abnormal cranial MRI 18/19	51/54 imaging - brainstem and cerebral white matter	Abnormal cranial MR- 35	-	MRI thrombosis, hyper-intensity in brain stem, pachymeningitis	Brain-stem, brainstem- plus isolated supratentorial location	Cascade sign in MRI brain
**Treatment**	High dose steroid 14	Steroid in 81%, AZR, CYC, Cyclosporine, IFX - 2 patients	Steroid in 55%, no treatment in two cases	-	All - steroid 3 - CYC, 2-AZR	Prednisolone use - Acute parenchymal- 66%, chronic progressive - 67%, CYC & IFX one patients with acute parenchymal	-	-	All steroid and AZA	All - steroids, CYC- 53 AZA- 40	High dose steroid, CYC, AZA, IFX

NBD: neuro-Behçet’s disease; CYC: Cyclophosphamide; IFX: Infliximab; AZA: Azathioprine.

## CONCLUSION

NBD is a rare complication associated with BD and there is limited literature on NBD in India. This case report presents the first documented case in India where parenchymal NBD presented as a stroke in a patient with chronic vascular BD despite the absence of typical skin or eye changes. However, the patient showed significant improvement in both neurological and vascular symptoms after receiving infliximab treatment. Early recognition and prompt treatment with medications can lead to a favourable response and improved outcomes. Further research and awareness of NBD are necessary to better understand its pathophysiology and optimise management strategies.
